# New Eco-Friendly 1-Alkyl-3-(4-phenoxybutyl) Imidazolium-Based Ionic Liquids Derivatives: A Green Ultrasound-Assisted Synthesis, Characterization, Antibacterial Activity and POM Analyses

**DOI:** 10.3390/molecules190811741

**Published:** 2014-08-07

**Authors:** Mouslim Messali, Mohamed R. Aouad, Wael S. El-Sayed, Adeeb Al-Sheikh Ali, Taibi Ben Hadda, Belkheir Hammouti

**Affiliations:** 1 Department of Chemistry, Taibah University, Al-Madina Al-Mounawara 30002, Saudi Arabia; 2 Laboratoire de Chimie & Electrochimie des Complexes Métalliques (LCECM) USTO-MB, University of Sciences and Technology Mohamed Boudiaf, BP 1505 Oran, El M'nouar, Algeria; 3 Microbiology department, Faculty of Science, Ain Shams University, Cairo 11566, Egypt; 4 Biology Department, Taibah University, Al-Madina Al-Mounawara 30002, Saudi Arabia; 5 Laboratoire de Chimie des Matériaux, Faculté des Sciences, Université Mohammed Premier, Oujda-60000, Morocco; 6 LCAE-URAC18, Faculté des Sciences, Université Mohammed Premier, B.P. 717, Oujda-60000, Morocco

**Keywords:** green procedure, ultrasound irradiation, ionic liquids, antimicrobial activity, Petra/Osiris/Molinspiration (POM) analyses

## Abstract

In view of the emerging importance of the ILs as “green” materials with wide applications and our general interests in green processes, a series of a twenty five new 1-alkyl-3-(4-phenoxybutyl) imidazolium-based ionic liquids (ILs) derivatives is synthesized using a facile and green ultrasound-assisted procedure. Their structures were characterized by FT-IR, ^1^H-NMR, ^13^C-NMR, ^11^B, ^19^F, ^31^P, and mass spectrometry. Antimicrobial screens of some selected ILs were conducted against a panel of Gram-positive and Gram-negative bacteria. The antimicrobial activity of each compound was measured by determination of the minimal inhibitory concentration (MIC) yielding very interesting and promising results. Their antibacterial activities are reported, and, on the basis of the experimental and virtual POM screening data available, attempt is also made to elucidate the structure activity relationship.

## 1. Introduction

Over the past two decades, Ionic liquids (ILs) have attracted considerable attention as friendly environmental substitutes for volatile organic solvents due to the several unique properties such as negligible vapor pressure, high thermal stability, easy recyclability, no flammability, and high ionic conductivity [1,2,3,4]. Generally, ILs are a group of low-melting-point salts containing organic cation, such as imidazolium, pyrrolidinium, or pyridazinium, paired with various anions, such as bromide or tetrafluoroborate [5].

Due to these unique properties, ILs have been widely synthesized and investigated as media for electrodeposition of metals [6,7,8], as a tool for lignocellulosic biomass fractionation [9], catalysis and biocatalysis [10,11,12,13,14,15,16], corrosion inhibition [17,18,19,20], food chemical science [21], and the nuclear industry [22].

Thus far, many chemists promoted to explore new procedures for the clean and efficient synthesis of ILs since the conventional syntheses of them are not benign [23]. Several modifications have been attempted including microwave irradiation, sonochemical reactions or solvent-free reactions. The use of these green technologies leads to many advantages, such as large reductions in reaction times, enhancements in conversions, sometimes in selectivity [24,25,26,27].

On the other hand, numerous studies have demonstrated the antimicrobial activity of various classes of ionic liquids against both environmental and clinically important microorganisms [28,29,30,31,32,33].

According to the above mentioned, and our ongoing research, interest in ionic liquids synthesis [34,35,36], we continued to combine the use of green technologies in the synthesis of new class of antimicrobial agents.

Some selected ILs were investigated for their anti-microbial activity against different pathogenic strains.

## 2. Results and Discussion

### 2.1. Chemistry

**Scheme 1:**
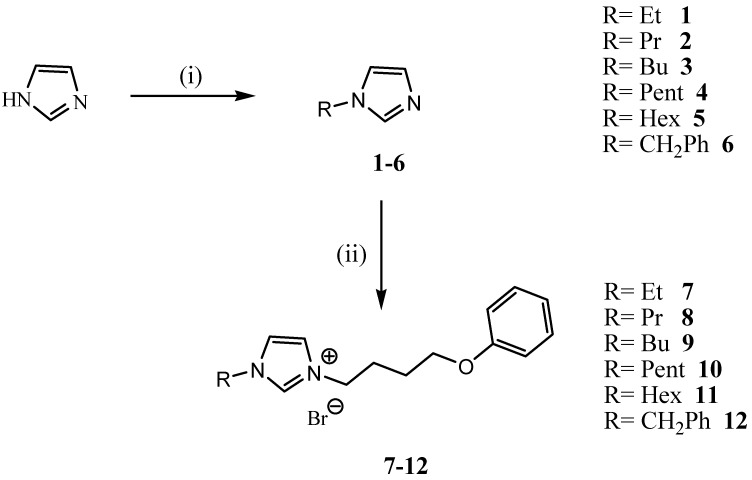
Synthesis of a variety of new imidazolium-based ionic liquids under both conventional and ultrasound irradiation methods.

**Scheme 2:**
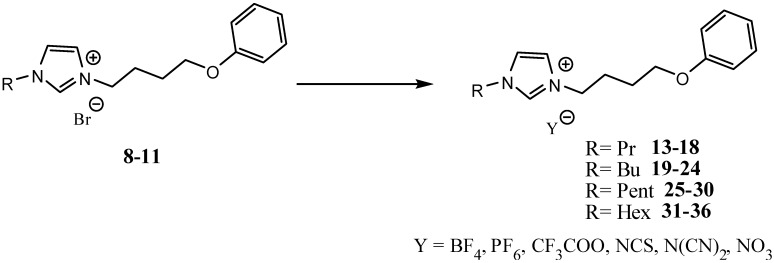
Anion metathesis using conventional preparation (CP) and ultrasonic irradiation conditions (US). (CP): MY, dichloromethane, 70 °C, 3 h; (US): dichloromethane, 70 °C, 45 min. M = Na, K.

In continuation of our previous work dealing with the development of novel room temperature functionalized ionic liquids [37], herein, we report the synthesis of a variety of new imidazolium-based ionic liquids under both conventional and ultrasound irradiation methods (Scheme 1 and Scheme 2).

(**i**) N-alkylation of imidazole: RBr, (KOH/K_2_CO_3_), Acetonitrile, 80 °C, 16 h. R = Et, Pr, Bu, Pent, Hex, Benzyl. (**ii**) *N*-alkylation of *N*-alkylimidazole: conventional preparation (CP) and ultrasonic irradiation conditions (US). (CP): PhO(CH_2_)_4_Br, toluene, 80 °C, 18 h; (US): toluene, 80 °C, 5 h.

Initially, various 1-alkyl-1H-imidazole **1**–**6** were easily prepared by treatment of imidazole with alkyl bromide with K_2_CO_3_/KOH in Acetonitrile. The nucleophilic alkylation of *N*-alkylelimidazoles **1**–**6** under standard conditions (toluene, 80 °C, 18 h), with different alkyl halides (1.1 eq) afforded the corresponding imidazolium halides in 78%–85% yield as oils after solvent removal by evaporation (Table 1).

**Table 1:** Reaction conditions and yields for the quaternization of N-alkylimidazole (**7**–**12**) using conventional preparation (CP) and ultrasound irradiation conditions (US).

Compound	R	Yield (%) of the Quaternization Step	
		CP_1_^a^	US ^b^
**7**	Et	79	85
**8**	Pr	80	87
**9**	Bu	78	89
**10**	Pent	79	88
**11**	Hex	80	87
**12**	CH_2_Ph	79	86

^a^ Time (18 h), Temperature (80 °C) in toluene; ^b^ Time (5 h), Temperature (80 °C) in toluene.

Previous work reports that the hydrophobicity of an ionic liquid increases with the length of the alkyl chain on the imidazolium ring, and this increase tends to raise viscosity [38,39]. This why six anions were used in the metathesis step in order to obtain low melting point and less viscous ILs.

The next step in the synthesis involved an anion exchange halides by using a slight excess of the anions sodium tetrafluoroborate, potassium hexafluorophosphate, trifluoroacetic acid sodium, sodium dicyanamide, sodium thiocyanate, or sodium nitrate anions (Scheme 2).

The resulting pure products from these reactions were subsequently obtained by filtration of the metal halide salts, followed by filtrate evaporation and washing of the residue with dichloromethane followed by further filtration to remove any remaining metal salts. Finally, evaporation of the filtrate afforded the desired ionic liquids **13**–**36** in good yields (Table 2). 

As already reported by our team, an anion exchange metathesis is easily performed by ultrasonic activation [34]. In a similar way, the preparation of ILs **13**–**36** was carried in a closed vessel and exposed to irradiation for 45 min at 70 °C using a sonication bath. The data presented in Table 2 indicated that very good yields were obtained in very short reaction times. As observed, the anion nature of the exchange agents did not affect product yields.

**Table 2:** Reaction conditions and yields for the anion metathesis reaction using conventional preparation (CP) and ultrasound irradiation conditions (US).

Compound	R	MY	Yield (%) for the Anion Metathesis	
			CP_2_^a^	(US) ^b^
**13**	Pr	NaBF_4_	95	96
**14**		KPF_6_	95	97
**15**		NaOOCCF_3_	92	98
**16**		NaN(CN)_2_	94	97
**17**		NaNCS	92	97
**18**		NaNO_3_	94	95
**19**	Bu	NaBF_4_	94	98
**20**		KPF_6_	93	97
**21**		NaOOCCF_3_	93	97
**22**		NaN(CN)_2_	92	96
**23**		NaNCS	93	95
**24**		NaNO_3_	92	96
**25**	Pent	NaBF_4_	95	98
**26**		KPF_6_	93	96
**27**		NaOOCCF_3_	95	97
**28**		NaN(CN)_2_	94	97
**29**		NaNCS	94	95
**30**		NaNO_3_	94	98
**31**	Hex	NaBF_4_	94	97
**32**		KPF_6_	93	96
**33**		NaOOCCF_3_	95	97
**34**		NaN(CN)_2_	92	96
**35**		NaNCS	93	95
**36**		NaNO_3_	94	97

^a^ Time (3 h), Temperature (70 °C) in acetonitrile; ^b^ Time (45 min), Temperature (70 °C) in acetonitrile.

The structures of all the newly synthesized ionic liquids were confirmed by ^1^H-NMR, ^13^C-NMR, ^11^B-NMR, ^19^F-NMR, ^31^P-NMR, FT-IR, and LCMS analysis. All spectroscopic data are detailed in the experimental part.

### 2.2. Antimicrobial Activity

The short generation times of bacteria compared with other living organisms give a good starting point to examine the toxicity of ILs [40]. This has indirectly led to the realization that some ILs exhibit anti-microbialactivity.

One of the objectives of the present study is therefore to investigate the anti-microbial activities of some synthesized ILs. For this, several types of human pathogens were selected to assess the potential toxicities of these ILs and theireffectiveness as anti-microbial agents.

In this aim, the water soluble ILs **7**–**12** were tested *in vitro* for their antibacterial activity against Gram-positive bacteria including; *Staphylococcus aureus*, *Streptococcus pneumonia*, *Bacillus subtilis* and *Bacillus cereus*, as well as Gram-negative bacteria, including *Escherichia coli*, *Klebsiella pneumoniae*, *Pseudomonas aeruginosa*, and *Acinetobacter baumannii*. These clinical isolates were selected based on their pathogenic properties. The antibacterial activity was measured by determination of MIC values in a range from 0 to 256 µg/mL and compared with those of some potent antibacterial compounds like mezlocillin, amikacin, tetracycline and nitrofurantion. MIC values are summarized (Table 3).

From the MIC values obtained, all compounds exhibited antibacterial activity with varying potential as well as spectrum. In general, all tested ILs (**7**–**12**) possessed congruent antibacterial activities against the growth of *E. coli*, *K. pneumoniae*, *S. aureus* and *S. pneumoniae* as compared with the standards mezlocillin, amikacin, tetracycline, and nitrofurantion while showed low activity (>128 µg/mL) against *P. aeruginosa* and *A. baumannii*.

**Table 3:** Antimicrobial activity of Ionic liquids **7**–**12** against eight bacterial strains.

Compounds	MIC (µg/mL)							
	*E. coli*	*K. pneumoniae*	*P. aeruginosa*	*A. baumannii*	*S. aureus*	*S. pneumoniae*	*B. subtilis*	*B. cereus*
**7**	64	64	>256	>256	64	64	128	>256
**8**	32	16	>256	>256	64	64	128	>256
**9**	16	16	>256	>256	32	16	128	>256
**10**	16	8	128	256	16	8	64	64
**11**	16	8	>256	128	8	8	16	32
**12**	16	32	>256	>256	64	32	64	>256
**Mezlocillin**	128	128	128	128	---	---	32	32
**Amikacin**	32	32	32	32	32	32	---	---
**Tetracycline**	16	16	---	16	---	8	4	4
**Nitrofurantion**	128	128	---	---	128	128	---	---

In particular, IL **11** exhibited the highest antibacterial activities in the series against most of the tested bacteria with MIC values (<32 µg/mL). Having MIC value of 8 for IL **11** against *K. pneumoniae*, *S. aureus*, and *S. pneumonia* makes it a good candidate as a potent antibacterial agent. Such relatively high antibacterial activity of IL **11** can be attributed to the presence of the hexyl chain which is absent in the IL **7** having lowest toxicity. This trend was also found in case of IL **9** and **10**. Therefore, it can be concluded that the biological activity of ILs **7**–**11** depends on the length of the alkyl chain and increased with increasing the hydrophobicity for the tested ILs.

Beside antibacterial activity, all tested ILs (**7**–**12**) showed broader spectrum revealed by detected antibacterial activity against Gram-positive and Gram-negative bacteria, particularly *S. aureus*, *S. pneumonia* and *E. coil*, *K. pneumonia*, respectively.

### 2.3. POM Analyses of Compounds **7**–**12**

For a molecule to be a potential drug, besides having a good biological activity, it must have good pharmacokinetic properties in biological systems. To access the pharmacokinetic profile of the synthesized molecules, we used well validated *in silico* tools: Osiris, Petra and Molinspiration. These tools have been validated with almost 7000 drug molecules available on the market.

The analysis of theoretical toxicity risks for the similar ILs using the Osiris program showed that similar ILs were less toxic than standard clinical drugsand can be used as therapeutic agents.

From the data evaluated in Table 4 indicates that, all structures are supposed to be non-mutagenic when run through the mutagenicity assessment system and, as far as irritating and reproductive effects are concerned, all the compounds are at low risk comparable with standard drugs used. The hydrophilicity character of each compound has been expressed in term of the log *p* value which correspond to the logarithm of its partition coefficient between n-octanol and water. It has been established that the absorption or permeation is greatly affected by the hydrophilicity (value of Log *p*). Accordingly, when Log *p* is higher than 5, the absorption or permeation decrease. 

On this basis, all the majority of compounds **7**–**12** is having log *P* values under the acceptable criteria should be active but they are not active because there is another crucial parameter which should be taken in consideration. It concerns the geometrical conformation of pharmacophore. The absorption, distribution characteristics and bioactivity was proved to be dependent to the geometrical parameter and the aqueous solubility of a compound. Consequently, the bad absorption could presumably due to the low solubility of the tested ILs. Further, the Table 4 shows drug-likeness of compounds **7**–**12** which is not in the comparable zone with that of standard drugs used.

We have calculated overall drug-score (DS) for the compounds **7**–**12** and compared with that of standard drugs (AMP, GENTA, and AMPHO) used as shown in Table 4 and Table 5. The DS combines drug-likeness, log *p*, log *S*, molecular weight, and toxicity risks, in one handy value that may be used to judge the compound’s overall potential to qualify for a drug. The reported compounds **7**–**12** showed low to moderate DS as compared with standard drugs used.

**Table 4:** Osiris calculations of toxicity risks and drug-score of compounds **7**–**12**. 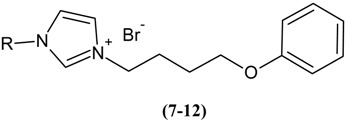

Compd.	R	Toxicity Risks ^[^^a]^				Drug-Score ^[^^b]^			
		MUT	TUMO	IRRI	REP	CLP	S	DL	DS
**7**	Ethyl	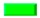	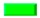	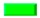	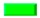	2.38	−2.67	−0.66	0.60
**8**	Propyl	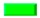	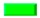	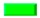	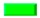	2.84	−2.94	−0.90	0.56
**9**	Butyl	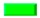	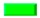	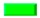	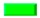	3.31	−3.21	−3.16	0.43
**10**	Pentyl	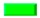	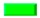	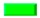	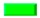	3.77	−3.48	−5.85	0.39
**11**	Hexyl	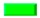	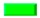	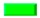	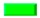	4.23	−3.76	−10.23	0.36
**12**	Benzyl	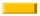	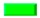	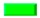	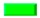	3.31	−3.69	−0.91	0.41
**Mezlocillin**	---	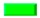	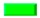	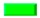	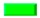	−0.03	−2.53	15.14	0.66
**Amikacin**	---	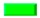	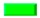	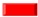	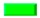	−8.00	−0.23	1.73	0.35
**Tetracycline**	---	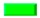	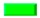	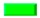	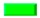	−1.02	−1.83	5.43	0.81
**Nitrofurantion**	---	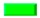	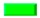	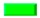	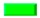	−0.07	−2.50	0.67	0.78

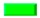
: not toxic; 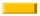: slightly toxic; 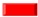: highly toxic. ^[^^a]^ MUT: mutagenic; TUMO: tumorigenic; IRRI: irritant; REP: reproductive effective. ^[^^b]^ CLP: cLogP, S: Solubility, DL: Druglikness, DS: Drug-Score.

### 2.4. Molinspiration Calculations

cLog *P* (octanol/water partition coefficient) is calculated by the methodology developed by Molinspiration as a sum of fragment based contributions and correction factors (Table 4). The method is very useful and can be applied to all organic and some organometallic molecules. Molecular Polar Surface Area values (TPSA) were also measured using the above mentioned methodology. O– and N– centred polar fragments are considered. PSA has been shown to be a useful parameter for determination of drug absorption and especially intestinal absorption, bioavailability, and blood–brain barrier penetration. Prediction results of compounds **7**–**12** molecular properties (TPSA, GPCR ligand, and ICM) are valued (Table 5).

**Table 5:** Molinspiration calculations of compounds **7**–**12**.

Compd.	MW(g/mol)	Physico-Chemical Properties ^[^^a]^				Drug Likeness ^[^^b]^					
		TPSA	O/NH	VIOL	VOL	GPC	ICM	KI	NRL	PI	EN
**7**	325	12.47	0	0	271	−0.06	−0.10	−0.18	−0.06	−0.22	0.01
**8**	339	12.47	0	0	287	0.04	−0.06	−0.13	0.03	−0.10	0.03
**9**	353	12.47	0	0	304	0.07	−0.06	−0.10	0.08	−0.05	0.05
**10**	367	12.47	0	0	321	0.10	−0.05	−0.07	0.12	0.01	0.07
**11**	381	12.47	0	0	338	0.12	−0.05	−0.05	0.13	0.03	0.06
**12**	387	12.47	0	0	325	0.09	−0.06	−0.01	0.08	0.04	0.04
**Mezlocillin**	539	173	3	2	434	−0.04	−0.43	−0.60	−0.58	0.66	0.10
**Amikacin**	585	332	17	3	510	0.32	−0.09	0.16	−0.10	0.78	0.45
**Tetracycline**	444	182	7	1	377	−0.15	−0.24	−0.53	−0.09	−0.04	0.52
**Nitrofurantion**	238	121	1	0	181	−1.36	−0.90	−1.21	−2.16	−1.45	−0.79

^[^^a]^ TPSA: Total polar surface area; O/NH: O---HN interaction; VIOL: number of violation; VOL: volume. ^[^^b]^ GPC: GPCR ligand; ICM: Ion channel modulator; KI: Kinase inhibitor; NRL: Nuclear receptor ligand; PI: Protease inhibitor; EI: Enzyme inhibitor.

The bioactivity properties were greatly affected by the electronic and steric factors. The obtained POM results confirm that most of these ILs could be used as potential antimicrobial activity after major modifications. Based on their great hydrosolubility properties, these compounds may be useful as solubilising substituents with potential anti-tumoralactivity. These results prompt several pertinent observations: (i) This type of ILs can furnish an interesting model for studying the interaction of target-antibiotics complexes with cancer phosphates because the possible charge (P=O^δ^---N^δ^^+^) modification of pi-charge pharmacophore group; (ii) The future flexiblelipophyl-hydrosoluble drugs containing Il moiety will enable us to prepare molecules for multi-therapeutic materials with high combined antibacterial/antiviral/antifungal/antitumoral activity.

## 3. Materials and Methods

### 3.1. Experimental

All new compounds were synthesized and characterized by ^1^H-NMR, ^13^C-NMR, IR, and LCMS. ^1^H-NMR (400 MHz) and ^13^C-NMR (100 MHz) spectra were measured in CDCl_3_ at room temperature. Chemical shifts (δ) were reported in ppm to a scale calibrated for tetramethylsilane (TMS), which is used as an internal standard. The LCMS spectra were measured with a Micromass, LCT mass spectrometer. IR spectra were recorded in NaCl or KBr disc on a Schimadzu 8201 PC, FTIR spectrophotometer (υ_max_ in cm^−1^). The ultrasound-assisted reactions were performed using a high intensity ultrasonic processor SUB Aqua 5 Plus-Grant with temperature controller (750 W), microprocessor controlled-2004, the ultrasonic frequency of the cleaning bath used equal 25 KHz.

### 3.2. Synthesis

General procedures for the synthesis of imidazolium halides (**7**–**12**) using conventional method. To the solution of *N*-alkylimidazole (1 eq) in toluene, was added alkyl bromide (1.1 eq) at room temperature, followed by stirring at 80 °C for 18 h. The completion of the reaction was marked by the separation of oil from the initially obtained clear and homogenous mixture of *N*-alkylimidazole and alkyl halide in toluene. The product was isolated by extraction to remove the unreacted starting materials and solvent. Subsequently, the imidazolium salt was washed with ethyl acetate. In each case, the IL/salt was finally dried at a reduced pressure to get rid of all the volatile organic compounds.

General procedure for the synthesis of imidazolium halides (**7**–**12**) using under Ultrasonic irradiation. Alkylimidazole (1 eq) and the appropriate alkyl bromide (1 eq) were placed in a closed vessel and exposed to irradiation for 5 h at 80 °C using a sonication bath. The product was then collected as described in the conventional procedure outlined earlier.

General procedure for the methathesis reaction of (**8**–**11**) leading to compounds (**13**–**36**) using conventional method. The quaternary salt (1 eq) was dissolved in dichloromethane to obtain a clear solution. To this solution of quaternary halide was added solution of sodium tetrafluoroborate, potassium hexafluorophosphate, trifluoroacetic acid sodium, sodium dicyanamide, sodium thiocyanate, or sodium nitrate (1.2 eq), followed by stirring at 70 °C for 3 h. The cooled reaction mixture was filtered through Celite to remove solid metal halide. The evaporation of dichloromethane leaded quantitatively to the desired ionic liquids.

General procedure for the ultrasound-assisted methathesis reaction of (**8**–**11**) leading to compounds (**13**–**36**). Imidazolium-halides salts (1 eq) and NaBF_4, _KPF_6_, CF_3_COONa, NaN(CN)_2_, NaNCS or NaNO_3_ (1 eq) were placed in a closed vessel and exposed to irradiation for 45 min at 70 °C using a sonication bath. The product was then collected as described in the conventional procedure outlined earlier.

### 3.3. Characterization

*1-Ethyl-3-(4-phenoxybutyl)-1H-imidazol-3-ium bromide* (**7**). ^1^H-NMR (CDCl_3_, 400 MHz): δ = 1.40 (t, *J* = 7.2 Hz, 3H), 1.69 (quint, *J* = 7.6 Hz, 2H), 1.96 (quint, *J* = 7.6 Hz, 2H), 3.96 (t, *J* = 7.6 Hz, 2H), 4.23 (q, *J* = 7.2 Hz, 2H), 4.30 (t, *J* = 7.6 Hz, 2H), 6.87–6.91 (m, 3H, Ar-H), 7.22–7.27 (m, 2H, Ar-H), 7.93–7.99 (m, 2H), 9.55 (s, 1H); ^13^C-NMR (CDCl_3_, 100 MHz): δ = 15.0 (CH_3_), 25.3 (CH_2_), 26.3 (CH_2_), 44.1 (CH_2_), 48.4 (CH_2_), 66.6 (CH_2_), 114.3 (CH), 120.4 (CH), 122.1 (CH), 122.3 (CH), 129.4 (CH), 135.8 (CH), 158.3 (C); IR (NaCl) υ_max _ 3132 (C-H Ar), 1599–1471 (C=C), 1165(C-N), 1082 (C-O) cm^−1^; LCMS (M^+^)-Br^−^ 245.2 found for C_15_H_21_N_2_O^+^. 

*1-Propyl-3-(4-phenoxybutyl)-1H-imidazol-3-ium bromide* (**8**). ^1^H-NMR (CDCl_3_, 400 MHz): δ = 0.88 (t, *J* = 7.2 Hz, 3H), 1.76 (quint, *J* = 7.6 Hz, 2H), 1.88 (sextet, *J* = 7.6 Hz, 2H), 2.07 (quint, *J* = 7.6 Hz, 2H), 3.93 (t, *J* = 7.6 Hz, 2H), 4.21 (t, *J* = 7.2 Hz, 2H), 4.40 (t, *J* = 7.6 Hz, 2H), 6.77–6.86 (m, 3H, Ar-H), 7.15–7.19 (m, 2H, Ar-H), 7.50–7.58 (m, 2H, Ar-H), 10.22 (s, 1H, Ar-H); ^13^C-NMR (CDCl_3_, 100 MHz): δ = 10.7 (CH_3_), 23.6 (CH_2_), 25.8 (CH_2_), 27.3 (CH_2_), 49.6 (CH_2_), 51.3 (CH_2_), 66.7 (CH_2_), 114.4 (CH), 120.8 (CH), 122.3 (CH), 122.4 (CH), 129.5 (CH), 136.6 (CH), 158.5 (C); IR (NaCl) υ_max _ 3131 (C-H Ar), 1599–1470 (C=C), 1164(C-N), 1083 (C-O) cm^−1^; LCMS (M^+^)-Br^−^ 259.2 found for C_16_H_23_N_2_O^+^.

*1-Butyl-3-(4-phenoxybutyl)-1H-imidazol-3-ium bromide* (**9**). ^1^H-NMR (CDCl_3_, 400 MHz): δ = 0.88 (t, *J* = 7.2 Hz, 3H), 1.31 (sextet, *J* = 7.6 Hz, 2H), 1.78–1.82 (m, *J* = 7.6 Hz, 4H), 2.10 (quint, *J* = 7.6 Hz, 2H), 3.94 (t, *J* = 7.6 Hz, 2H), 4.25 (t, *J* = 7.2 Hz, 2H), 4.41 (t, *J* = 7.6 Hz, 2H), 6.79–6.86 (m, 3H, Ar-H), 7.17–7.21 (m, 2H, Ar-H), 7.47–7.58 (m, 2H, Ar-H), 10.22 (s, 1H, Ar-H); ^13^C-NMR (CDCl_3_, 100 MHz): δ = 13.4 (CH_3_), 19.4 (CH_2_), 25.8 (CH_2_), 27.3 (CH_2_), 32.1 (CH_2_), 48.7 (CH_2_), 49.6 (CH_2_), 66.8 (CH_2_), 114.4 (CH), 120.8 (CH), 122.2 (CH), 122.4 (CH), 129.5 (CH), 136.6 (CH), 158.5 (C); IR (NaCl) υ_max_ 3133 (C-H Ar), 1600–1471 (C=C), 1166(C-N), 1082 (C-O) cm^−1^; LCMS (M^+^)-Br^−^ 273.3 found for C_17_H_25_N_2_O^+^.

*1-Pentyl-3-(4-phenoxybutyl)-1H-imidazol-3-ium bromide* (**10**). ^1^H-NMR (CDCl_3_, 400 MHz): δ = 0.67 (t, *J* = 7.2 Hz, 3H), 1.04–1.15 (m, 4H), 1.62–1.72 (m, 4H), 1.97 (quint, *J* = 7.6 Hz, 2H), 3.79 (t, *J* = 7.6 Hz, 2H), 4.11 (t, *J* = 7.2 Hz, 2H), 4.28 (t, *J* = 7.6 Hz, 2H), 6.79–6.86 (m, 3H, Ar-H), 7.65–7.71 (m, 2H, Ar-H), 7.02–7.06 (m, 2H, Ar-H), 10.13 (s, 1H, Ar-H); ^13^C-NMR (CDCl_3_, 100 MHz): δ = 13.7 (CH_3_), 21.8 (CH_2_), 25.7 (CH_2_), 27.2 (CH_2_), 28.0 (CH_2_), 29.7 (CH_2_), 49.4 (CH_2_), 49.8 (CH_2_), 66.6 (CH_2_), 114.2 (CH), 120.6 (CH), 122.2 (CH), 122.5 (CH), 129.3 (CH), 136.3 (CH), 158.4 (C); IR (NaCl) υ_max_ 3132 (C-H Ar), 1600–1471 (C=C), 1164 (C-N), 1081 (C-O) cm^−1^; LCMS (M^+^)-Br^−^ 287.3 found for C_18_H_27_N_2_O^+^.

*1-Hexyl-3-(4-phenoxybutyl)-1H-imidazol-3-ium bromide* (**11**). ^1^H-NMR (CDCl_3_, 400 MHz): δ = 0.94 (t, *J* = 7.2 Hz, 3H), 1.14 (quint, *J* = 7.6 Hz, 2H), 1.39–1.41 (m, 4H), 1.69 (quint, *J* = 7.6 Hz, 2H), 1.77 (quint, *J* = 7.6 Hz, 2H), 1.96 (quint, *J* = 7.6 Hz, 2H), 3.96 (t, *J* = 7.6 Hz, 2H), 4.27 (quint, *J* = 7.2 Hz, 2H), 4.46 (t, *J* = 7.6 Hz, 2H), 6.87–6.91 (m, 3H, Ar-H), 7.22–7.27 (m, 2H, Ar-H), 7.93–7.99 (m, 2H, Ar-H), 9.55 (s, 1H, Ar-H); ^13^C-NMR (CDCl_3_, 100 MHz): δ = 13.9 (CH_3_), 22.6 (CH_2_), 24.8 (CH_2_), 27.2 (CH_2_), 27.3 (CH_2_), 32.6 (CH_2_), 33.9 (CH_2_), 44.1 (CH_2_), 48.3 (CH_2_), 66.6 (CH_2_), 114.3 (CH), 120.4 (CH), 122.1 (CH), 122.3 (CH), 129.4 (CH), 135.8 (CH), 158.3 (C); IR (NaCl) υ_max_ 3132 (C-H Ar), 1598–1472 (C=C), 1165 (C-N), 1081 (C-O) cm^−1^; LCMS (M^+^)-Br^−^ 301.3 found for C_19_H_29_N_2_O^+^.

*1-Benzyl-3-(4-phenoxybutyl)-1H-imidazol-3-ium bromide* (**12**). White crystals, Mp 153–155 °C_,_^1^H-NMR (CDCl_3_, 400 MHz): δ = 1.77 (quint, *J* = 8, 2H), 2.07 (quint, *J* = 8 Hz, 2H), 3.91 (t, *J* = 8 Hz, 2H), 4.35 (t, *J* = 8 Hz, 2H), 5.52 (s, 2H), 6.77–6.88 (m, 3H), 7.17–7.55 (m, 9H), 10.45 (s, 1H); ^13^C-NMR (CDCl_3_, 100 MHz): δ = 25.7 (CH_2_), 26.9 (CH_2_), 49.7 (CH_2_), 53.2 (CH_2_), 66.7 (CH_2_), 114.4 (CH), 120.8 (CH), 122.2 (CH), 122.6 (CH), 128.9 (CH), 129.4 (CH), 129.5 (CH), 133.0 (C), 135.5 (CH), 158.6 (C); IR (KBr) υ_max_ 3130 (C-H Ar), 1599–1471 (C=C), 1164 (C-N), 1081 (C-O) cm^−1^; LCMS (M^+^)-Br^−^ 307.3.3 found for C_20_H_23_N_2_O^+^.

*1-Propyl-3-(4-phenoxybutyl)-1H-imidazol-3-ium tetrafluoroborate* (**13**). ^1^H-NMR (CDCl_3_, 400 MHz): δ = 0.88 (t, *J* = 7.2 Hz, 3H), 1.76 (quint, *J* = 7.6 Hz, 2H), 1.88 (sextet, *J* = 7.6 Hz, 2H), 2.07 (quint, *J* = 7.6 Hz, 2H), 3.93 (t, *J* = 7.6 Hz, 2H), 4.21 (t, *J* = 7.2 Hz, 2H), 4.40 (t, *J* = 7.6 Hz, 2H), 6.77–6.86 (m, 3H, Ar-H), 7.15–7.19 (m, 2H, Ar-H), 7.50–7.58 (m, 2H, Ar-H), 10.22 (s, 1H, Ar-H); ^13^C-NMR (CDCl_3_, 100 MHz): δ = 8.7 (CH_3_), 21.6 (CH_2_), 24.0 (CH_2_), 25.3 (CH_2_), 47.9 (CH_2_), 49.6 (CH_2_), 65.0 (CH_2_), 112.6 (CH), 119.0 (CH), 120.7 (CH), 122.2 (CH), 127.7 (CH), 133.7 (CH), 158.5 (C); ^19^F-NMR (CDCl_3, _376.5 MHz): δ = −150.65; ^11^B-NMR (CDCl_3, _128 MHz): δ = −0.96; IR (NaCl) υ_max_ 3133 (C-H Ar), 1598–1472 (C=C), 1166 (C-N), 1084 (C-O) cm^−1^; LCMS (M^+^)-BF_4_^−^ 259.2 found for C_16_H_23_N_2_O^+^.

*1-Propyl-3-(4-phenoxybutyl)-1H-imidazol-3-ium hexafluorophospate* (**14**). ^1^H-NMR (CDCl_3_, 400 MHz): δ = 0.94 (t, *J* = 7.2 Hz, 3H), 1.80 (quint, *J* = 7.6 Hz, 2H), 1.87 (sextet, *J* = 7.6 Hz, 2H), 2.06 (quint, *J* = 7.6 Hz, 2H), 3.98 (t, *J* = 7.6 Hz, 2H), 4.10 (t, *J* = 7.2 Hz, 2H), 4.25 (t, *J* = 7.6 Hz, 2H), 6.86–6.96 (m, 3H, Ar-H), 7.25–7.32 (m, 5H, Ar-H ), 8.58 (s, 1H, Ar-H); ^13^C-NMR (CDCl_3_, 100 MHz): δ = 10.5 (CH_3_), 23.2 (CH_2_), 25.7 (CH_2_), 27.0 (CH_2_), 49.8 (CH_2_), 51.6 (CH_2_), 66.7 (CH_2_), 114.4 (CH), 120.9 (CH), 122.2 (CH), 122.3 (CH), 129.6 (CH), 135.2 (CH), 158.6 (C); ^19^F-NMR (CDCl_3, _376.5 MHz): δ = −71.10 ; ^31^P NMR (CDCl_3, _162 MHz): δ = −144.31 (sep, *J =* 712.8 Hz); IR (NaCl) υ_max _3132 (C-H Ar), 1599–1471 (C=C), 1165(C-N), 1082 (C-O) cm^−1^; LCMS (M^+^)-PF_6_^−^ 259.2 found for C_16_H_23_N_2_O^+^.

*1-Propyl-3-(4-phenoxybutyl)-1H-imidazol-3-ium trifluroacetate* (**15**). ^1^H-NMR (CDCl_3_, 400 MHz): δ = 0.88 (t, *J* = 7.2 Hz, 3H), 1.75 (quint, *J* = 7.6 Hz, 2H), 1.83 (sextet, *J* = 7.6 Hz, 2H), 2.03 (quint, *J* = 7.6 Hz, 2H), 3.92 (t, *J* = 7.6 Hz, 2H), 4.14 (t, *J* = 7.2 Hz, 2H), 4.33 (t, *J* = 7.6 Hz, 2H), 6.79–6.88 (m, 3H, Ar-H), 7.18–7.22 (m, 3H, Ar-H ), 7.40–7.47 (m, 2H, Ar-H ), 10.10 (s, 1H, Ar-H); ^13^C-NMR (CDCl_3_, 100 MHz): δ = 10.5 (CH_3_), 23.4 (CH_2_), 25.7 (CH_2_), 27.2 (CH_2_), 49.5 (CH_2_), 51.3 (CH_2_), 66.7 (CH_2_), 114.3 (CH), 120.8 (CH), 122.1 (CH), 122.2 (CH), 129.4 (CH), 137.0 (CH), 158.5 (C); ^19^F-NMR (CDCl_3, _376.5 MHz): δ = −75.42; IR (NaCl) υ_max _3131 (C-H Ar), 1599–1469 (C=C), 1166 (C-N), 1079 (C-O) cm^−1^; LCMS (M^+^)-CF_3_CO_2_^−^ 259.2 found for C_16_H_23_N_2_O^+^.

*1-Propyl-3-(4-phenoxybutyl)-1H-imidazol-3-ium dicyanoamine* (**16**). ^1^H-NMR (CDCl_3_, 400 MHz): δ = 0.91 (t, *J* = 7.2 Hz, 3H), 1.79 (quint, *J* = 7.6 Hz, 2H), 1.82 (sextet, *J* = 7.6 Hz, 2H), 2.08 (quint, *J* = 7.6 Hz, 2H), 3.95 (t, *J* = 7.6 Hz, 2H), 4.19 (t, *J* = 7.2 Hz, 2H), 4.36 (t, *J* = 7.6 Hz, 2H), 6.81–6.88 (m, 3H, Ar-H), 7.18–7.22 (m, 3H, Ar-H ), 7.45–7.51 (m, 2H, Ar-H ), 9.28 (s, 1H, Ar-H); ^13^C-NMR (CDCl_3_, 100 MHz): δ = 8.8 (CH_3_), 21.6 (CH_2_), 24.0 (CH_2_), 25.4 (CH_2_), 48.1 (CH_2_), 49.8 (CH_2_), 64.9 (CH_2_), 112.6 (CH), 118.9 (CH), 120.6 (CH), 120.7 (CH), 127.6 (CH), 134.0 (CH), 156.6 (C); IR (NaCl) υ_max _3133 (C-H Ar), 1598–1472 (C=C), 1163 (C-N), 1080 (C-O) cm^−1^; LCMS (M^+^)-(CN)_2_N^−^ 259.2 found for C_16_H_23_N_2_O^+^.

*1-Propyl-3-(4-phenoxybutyl)-1H-imidazol-3-ium thiocyanate* (**17**). ^1^H-NMR (CDCl_3_, 400 MHz): δ = 0.89 (t, *J* = 7.2 Hz, 3H), 1.78 (quint, *J* = 7.6 Hz, 2H), 1.86 (sextet, *J* = 7.6 Hz, 2H), 2.03 (quint, *J* = 7.6 Hz, 2H), 3.93 (t, *J* = 7.6 Hz, 2H), 4.12 (t, *J* = 7.2 Hz, 2H), 4.29 (t, *J* = 7.6 Hz, 2H), 6.79–6.87 (m, 3H, Ar-H), 7.16–7.22 (m, 3H, Ar-H ), 7.39–7.46 (m, 2H, Ar-H ), 9.50 (s, 1H, Ar-H); ^13^C-NMR (CDCl_3_, 100 MHz): δ = 8.8 (CH_3_), 21.6 (CH_2_), 23.9 (CH_2_), 25.3 (CH_2_), 47.9 (CH_2_), 49.6 (CH_2_), 64.8 (CH_2_), 112.5 (CH), 119.0 (CH), 120.5 (CH), 120.6 (CH), 127.6 (CH), 134.1 (CH), 156.6 (C); IR (NaCl) υ_max_ 3129 (C-H Ar), 1597–1469 (C=C), 1163 (C-N), 1079 (C-O) cm^−1^; LCMS (M^+^)-SCN^−^ 259.2 found for C_16_H_23_N_2_O^+^.

*1-Propyl-3-(4-phenoxybutyl)-1H-imidazol-3-ium nitrate* (**18**). ^1^H-NMR (CDCl_3_, 400 MHz): δ = 0.75 (t, *J* = 7.2 Hz, 3H), 1.63 (quint, *J* = 7.6 Hz, 2H), 1.74 (sextet, *J* = 7.6 Hz, 2H), 1.94 (quint, *J* = 7.6 Hz, 2H), 3.79 (t, *J* = 7.6 Hz, 2H), 4.07 (t, *J* = 7.2 Hz, 2H), 4.23 (t, *J* = 7.6 Hz, 2H), 6.66–6.74 (m, 3H, Ar-H), 7.03–7.07 (m, 3H, Ar-H ), 7.42–7.50 (m, 2H, Ar-H ), 9.92 (s, 1H, Ar-H); ^13^C-NMR (CDCl_3_, 100 MHz): δ = 10.6 (CH_3_), 23.4 (CH_2_), 25.7 (CH_2_), 27.1 (CH_2_), 49.4 (CH_2_), 51.2 (CH_2_), 66.6 (CH_2_), 114.3 (CH), 120.7 (CH), 122.3 (CH), 122.4 (CH), 129.3 (CH), 136.4 (CH), 156.4 (C); IR (NaCl) υ_max_ 3132 (C-H Ar), 1598–1470 (C=C), 1165 (C-N), 1081 (C-O) cm^−1^; LCMS (M^+^)-NO_3_^−^ 259.2 found for C_16_H_23_N_2_O^+^.

*1-Butyl-3-(4-phenoxybutyl)-1H-imidazol-3-ium tetrafluoroborate* (**19**). ^1^H-NMR (CDCl_3_, 400 MHz): δ = 0.90 (t, *J* = 7.2 Hz, 3H), 1.33 (sextet, *J* = 7.6 Hz, 2H), 1.80–1.84 (m, *J* = 7.6 Hz, 4H), 2.12 (quint, *J* = 7.6 Hz, 2H), 3.96 (t, *J* = 7.6 Hz, 2H), 4.27 (t, *J* = 7.2 Hz, 2H), 4.43 (t, *J* = 7.6 Hz, 2H), 6.81–6.89 (m, 3H, Ar-H), 7.19–7.24 (m, 2H, Ar-H), 7.50–7.61 (m, 2H, Ar-H), 9.58 (s, 1H, Ar-H); ^13^C-NMR (CDCl_3_, 100 MHz): δ = 12.4 (CH_3_), 18.4 (CH_2_), 24.8 (CH_2_), 26.3 (CH_2_), 31.1 (CH_2_), 47.6 (CH_2_), 48.7 (CH_2_), 65.8 (CH_2_), 113.4 (CH), 119.8 (CH), 121.2 (CH), 121.4 (CH), 128.5 (CH), 135.6 (CH), 157.5 (C); ^19^F-NMR (CDCl_3, _376.5 MHz): δ = −150.35; ^11^B-NMR (CDCl_3, _128 MHz): δ = −0.98; IR (NaCl) υ_max_ 3130 (C-H Ar), 1599–1471 (C=C), 1164 (C-N), 1082 (C-O) cm^−1^; LCMS (M^+^)-BF_4_^−^ 273.3 found for C_17_H_25_N_2_O^+^.

*1-Butyl-3-(4-phenoxybutyl)-1H-imidazol-3-ium hexafluorophospate* (**20**). ^1^H-NMR (CDCl_3_, 400 MHz): δ = 0.87 (t, *J* = 7.2 Hz, 3H), 1.30 (sextet, *J* = 7.6 Hz, 2H), 1.77–1.81 (m, *J* = 7.6 Hz, 4H), 2.09 (quint, *J* = 7.6 Hz, 2H), 3.92 (t, *J* = 7.6 Hz, 2H), 4.23 (t, *J* = 7.2 Hz, 2H), 4.39 (t, *J* = 7.6 Hz, 2H), 6.77–6.86 (m, 3H, Ar-H), 7.15–7.20 (m, 2H, Ar-H), 7.46–7.57 (m, 2H, Ar-H), 8.78 (s, 1H, Ar-H); ^13^C-NMR (CDCl_3_, 100 MHz): δ = 12.8 (CH_3_), 18.8 (CH_2_), 25.2 (CH_2_), 26.8 (CH_2_), 31.5 (CH_2_), 48.0 (CH_2_), 49.1 (CH_2_), 66.2 (CH_2_), 113.7 (CH), 120.2 (CH), 121.6 (CH), 121.6 (CH), 128.9 (CH), 136.0 (CH), 158.2 (C); ^19^F-NMR (CDCl_3, _376.5 MHz): δ = −72.96; ^31^P NMR (CDCl_3, _162 MHz): δ = −144.96 (sep, *J =* 712.8 Hz); IR (NaCl) υ_max _3134 (C-H Ar), 1598–1469 (C=C), 1163 (C-N), 1080 (C-O) cm^−1^; LCMS (M^+^)-PF_6_^−^ 273.3 found for C_17_H_25_N_2_O^+^.

*1-Butyl-3-(4-phenoxybutyl)-1H-imidazol-3-ium trifluroacetate* (**21**). ^1^H-NMR (CDCl_3_, 400 MHz): δ = 0.88 (t, *J* = 7.2 Hz, 3H), 1.32 (sextet, *J* = 7.6 Hz, 2H), 1.78-1.83 (m, *J* = 7.6 Hz, 4H), 2.10 (quint, *J* = 7.6 Hz, 2H), 3.94 (t, *J* = 7.6 Hz, 2H), 4.24 (t, *J* = 7.2 Hz, 2H), 4.41 (t, *J* = 7.6 Hz, 2H), 6.78–6.88 (m, 3H, Ar-H), 7.16–7.22 (m, 2H, Ar-H), 7.47–7.59 (m, 2H, Ar-H), 8.99 (s, 1H, Ar-H); ^13^C-NMR (CDCl_3_, 100 MHz): δ = 13.1 (CH_3_), 19.1 (CH_2_), 25.5 (CH_2_), 27.1 (CH_2_), 31.8 (CH_2_), 48.3 (CH_2_), 49.4 (CH_2_), 66.5 (CH_2_), 114.0 (CH), 120.6 (CH), 121.9 (CH), 121.9 (CH), 129.2 (CH), 136.3 (CH), 158.5 (C); ^19^F-NMR (CDCl_3, _376.5 MHz): δ = −75.72;IR (NaCl) υ_max _3132 (C-H Ar), 1599–1471 (C=C), 1165(C-N), 1082 (C-O) cm^−1^; LCMS (M^+^)-CF_3_CO_2_^−^ 273.3 found for C_17_H_25_N_2_O^+^.

*1-Butyl-3-(4-phenoxybutyl)-1H-imidazol-3-ium dicyanoamine* (**22**). ^1^H-NMR (CDCl_3_, 400 MHz): δ = 0.88 (t, *J* = 7.2 Hz, 3H), 1.31 (sextet, *J* = 7.6 Hz, 2H), 1.78–1.82 (m, *J* = 7.6 Hz, 4H), 2.10 (quint, *J* = 7.6 Hz, 2H), 3.94 (t, *J* = 7.6 Hz, 2H), 4.25 (t, *J* = 7.2 Hz, 2H), 4.41 (t, *J* = 7.6 Hz, 2H), 6.79–6.86 (m, 3H, Ar-H), 7.17–7.21 (m, 2H, Ar-H), 7.47–7.58 (m, 2H, Ar-H), 10.22 (s, 1H, Ar-H); ^13^C-NMR (CDCl_3_, 100 MHz): δ = 13.4 (CH_3_), 19.4 (CH_2_), 25.8 (CH_2_), 27.3 (CH_2_), 32.1 (CH_2_), 48.7 (CH_2_), 49.6 (CH_2_), 66.9 (CH_2_), 114.5 (CH), 120.8 (CH), 122.2 (CH), 122.4 (CH), 129.5 (CH), 136.6 (CH), 158.5 (C); IR (NaCl) υ_max _3130 (C-H Ar), 1600–1470 (C=C), 1164 (C-N), 1081 (C-O) cm^−1^; LCMS (M^+^)-(CN)_2_N^ −^ 273.3 found for C_17_H_25_N_2_O^+^.

*1-Butyl-3-(4-phenoxybutyl)-1H-imidazol-3-ium thiocyanate* (**23**). ^1^H-NMR (CDCl_3_, 400 MHz): δ = 0.92 (t, *J* = 7.2 Hz, 3H), 1.35 (sextet, *J* = 7.6 Hz, 2H), 1.82–1.86 (m, *J* = 7.6 Hz, 4H), 2.13 (quint, *J* = 7.6 Hz, 2H), 3.97 (t, *J* = 7.6 Hz, 2H), 4.28 (t, *J* = 7.2 Hz, 2H), 4.45 (t, *J* = 7.6 Hz, 2H), 6.79–6.86 (m, 3H, Ar-H), 7.17–7.21 (m, 2H, Ar-H), 7.47–7.58 (m, 2H, Ar-H), 8.99 (s, 1H, Ar-H); ^13^C-NMR (CDCl_3_, 100 MHz): δ = 13.4 (CH_3_), 19.4 (CH_2_), 25.8 (CH_2_), 27.3 (CH_2_), 32.1 (CH_2_), 48.7 (CH_2_), 49.6 (CH_2_), 66.8 (CH_2_), 114.4 (CH), 120.8 (CH), 122.2 (CH), 122.4 (CH), 129.5 (CH), 136.6 (CH), 158.5 (C); IR (NaCl) υ_max _3128 (C-H Ar), 1599–1471 (C=C), 1162 (C-N), 1079 (C-O) cm^−1^; LCMS (M^+^)-SCN^−^ 273.3 found for C_17_H_25_N_2_O^+^.

*1-Butyl-3-(4-phenoxybutyl)-1H-imidazol-3-ium nitrate* (**24**). ^1^H-NMR (CDCl_3_, 400 MHz): δ = 0.90 (t, *J* = 7.2 Hz, 3H), 1.33 (sextet, *J* = 7.6 Hz, 2H), 1.80–1.84 (m, *J* = 7.6 Hz, 4H), 2.12 (quint, *J* = 7.6 Hz, 2H), 3.96 (t, *J* = 7.6 Hz, 2H), 4.27 (t, *J* = 7.2 Hz, 2H), 4.43 (t, *J* = 7.6 Hz, 2H), 6.81–6.89 (m, 3H, Ar-H), 7.19–7.24 (m, 2H, Ar-H), 7.50–7.61 (m, 2H, Ar-H), 8.89 (s, 1H, Ar-H); ^13^C-NMR (CDCl_3_, 100 MHz): δ = 13.0 (CH_3_), 19.1 (CH_2_), 25.4 (CH_2_), 27.1 (CH_2_), 31.7 (CH_2_), 48.3 (CH_2_), 49.3 (CH_2_), 66.5 (CH_2_), 113.9 (CH), 120.6 (CH), 121.8 (CH), 121.9 (CH), 129.1 (CH), 136.2 (CH), 158.5 (C); IR (NaCl) υ_max _3131 (C-H Ar), 1598–1471 (C=C), 1163 (C-N), 1083 (C-O) cm^−1^; LCMS (M^+^)-NO_3_^−^ 273.3 found for C_17_H_25_N_2_O^+^.

*1-Pentyl-3-(4-phenoxybutyl)-1H-imidazol-3-ium tetrafluoroborate* (**25**). ^1^H-NMR (CDCl_3_, 400 MHz): δ = 0.87 (t, *J* = 7.2 Hz, 3H), 1.30–1.32 (m, 4H), 1.80–1.83 (m, 4H), 2.08 (quint, *J* = 7.6 Hz, 2H), 3.97 (t, *J* = 7.6 Hz, 2H), 4.15 (t, *J* = 7.2 Hz, 2H), 4.28 (t, *J* = 7.6 Hz, 2H), 6.85–6.92 (m, 3H, Ar-H), 7.23–7.27 (m, 2H, Ar-H), 7.35–7.42 (m, 2H, Ar-H), 8.90 (s, 1H, Ar-H); ^13^C-NMR (CDCl_3_, 100 MHz): δ = 11.9 (CH_3_), 20.1 (CH_2_), 23.9 (CH_2_), 25.3 (CH_2_), 26.3 (CH_2_), 27.8 (CH_2_), 47.9 (CH_2_), 48.2 (CH_2_), 64.9 (CH_2_), 112.6 (CH), 119.0 (CH), 120.5 (CH), 120.6 (CH), 127.7 (CH), 133.8 (CH), 156.8 (C); ^19^F-NMR (CDCl_3, _376.5 MHz): δ = −151.03; ^11^B-NMR (CDCl_3, _128 MHz): δ = −0.93; IR (NaCl) υ_max _3135 (C-H Ar), 1601–1475 (C=C), 1165 (C-N), 1081 (C-O) cm^−1^; LCMS (M^+^)-BF_4_^−^ 287.3 found for C_18_H_27_N_2_O^+^.

*1-Pentyl-3-(4-phenoxybutyl)-1H-imidazol-3-ium hexafluorophospate* (**26**). ^1^H-NMR (CDCl_3_, 400 MHz): δ = 0.78 (t, *J* = 7.2 Hz, 3H), 1.21–1.23 (m, 4H), 1.67–1.75 (m, 4H), 1.95 (quint, *J* = 7.6 Hz, 2H), 3.86 (t, *J* = 7.6 Hz, 2H), 4.02 (t, *J* = 7.2 Hz, 2H), 4.13 (t, *J* = 7.6 Hz, 2H), 6.76–6.85 (m, 3H, Ar-H), 7.14–7.25 (m, 4H, Ar-H), 8.42 (s, 1H, Ar-H); ^13^C-NMR (CDCl_3_, 100 MHz): δ = 12.0 (CH_3_), 20.2 (CH_2_), 24.0 (CH_2_), 25.2 (CH_2_), 26.4 (CH_2_), 27.8 (CH_2_), 48.0 (CH_2_), 48.3 (CH_2_), 65.0 (CH_2_), 112.7 (CH), 119.1 (CH), 120.6 (CH), 120.7 (CH), 127.8 (CH), 133.2 (CH), 156.9 (C); ^19^F-NMR (CDCl_3, _376.5 MHz): δ = −70.94 ; ^31^P NMR (CDCl_3, _162 MHz): δ = −144.26 (sep*, J =* 712.8 Hz); IR (NaCl) υ_max _3132 (C-H Ar), 1599–1469 (C=C), 1167 (C-N), 1084 (C-O) cm^−1^; LCMS (M^+^)-PF_6_^−^ 287.3 found for C_18_H_27_N_2_O^+^.

*1-Pentyl-3-(4-phenoxybutyl)-1H-imidazol-3-ium trifluroacetate* (**27**). ^1^H-NMR (CDCl_3_, 400 MHz): δ = 0.82 (t, *J* = 7.2 Hz, 3H), 1.21–1.29 (m, 4H), 1.74–1.82 (m, 4H), 2.05 (quint, *J* = 7.6 Hz, 2H), 3.93 (t, *J* = 7.6 Hz, 2H), 4.18 (t, *J* = 7.2 Hz, 2H), 4.34 (t, *J* = 7.6 Hz, 2H), 6.80–6.89 (m, 3H, Ar-H), 7.18–7.22 (m, 2H, Ar-H), 7.38–7.48 (m, 2H, Ar-H), 10.02 (s, 1H, Ar-H); ^13^C-NMR (CDCl_3_, 100 MHz): δ = 11.9 (CH_3_), 20.1 (CH_2_), 23.9 (CH_2_), 25.4 (CH_2_), 26.3 (CH_2_), 28.0 (CH_2_), 47.7 (CH_2_), 48.1 (CH_2_), 64.9 (CH_2_), 112.5 (CH), 119.0 (CH), 120.2 (CH), 120.5 (CH), 127.6 (CH), 135.1 (CH), 156.7 (C); ^19^F-NMR (CDCl_3, _376.5 MHz): δ = −75.36 ; IR (NaCl) υ_max _3135 (C-H Ar), 1599–1473 (C=C), 1165 (C-N), 1082 (C-O) cm^−1^; LCMS (M^+^)-CF_3_CO_2_^−^ 287.3 found for C_18_H_27_N_2_O^+^.

*1-Pentyl-3-(4-phenoxybutyl)-1H-imidazol-3-ium dicyanoamine* (**28**). ^1^H-NMR (CDCl_3_, 400 MHz): δ = 0.82 (t, *J* = 7.2 Hz, 3H), 1.21–1.30 (m, 4H), 1.75–1.84 (m, 4H), 2.06 (quint, *J* = 7.6 Hz, 2H), 3.94 (t, *J* = 7.6 Hz, 2H), 4.15 (t, *J* = 7.2 Hz, 2H), 4.30 (t, *J* = 7.6 Hz, 2H), 6.79–6.87 (m, 3H, Ar-H), 7.17–7.21 (m, 2H, Ar-H), 7.38–7.47 (m, 2H, Ar-H), 9.56 (s, 1H, Ar-H); ^13^C-NMR (CDCl_3_, 100 MHz): δ = 11.9 (CH_3_), 20.0 (CH_2_), 23.9 (CH_2_), 25.4 (CH_2_), 26.3 (CH_2_), 27.8 (CH_2_), 47.9 (CH_2_), 48.2 (CH_2_), 64.8 (CH_2_), 112.5 (CH), 119.0 (CH), 120.5 (CH), 120.6 (CH), 127.6 (CH), 134.1 (CH), 156.6 (C); IR (NaCl) υ_max _3129 (C-H Ar), 1598–1471 (C=C), 1163 (C-N), 1079 (C-O) cm^−1^; LCMS (M^+^)-(CN)_2_N^−^ 287.3 found for C_18_H_27_N_2_O^+^.

*1-Pentyl-3-(4-phenoxybutyl)-1H-imidazol-3-ium thiocyanate* (**29**). ^1^H-NMR (CDCl_3_, 400 MHz): δ = 0.85 (t, *J* = 7.2 Hz, 3H), 1.24–1.33 (m, 4H), 1.78–1.87 (m, 4H), 2.09 (quint, *J* = 7.6 Hz, 2H), 3.97 (t, *J* = 7.6 Hz, 2H), 4.18 (t, *J* = 7.2 Hz, 2H), 4.33 (t, *J* = 7.6 Hz, 2H), 6.82–6.90 (m, 3H, Ar-H), 7.20–7.24 (m, 2H, Ar-H), 7.41–7.50 (m, 2H, Ar-H), 9.59 (s, 1H, Ar-H); ^13^C-NMR (CDCl_3_, 100 MHz): δ = 11.8 (CH_3_), 19.9 (CH_2_), 23.8 (CH_2_), 25.3 (CH_2_), 26.2 (CH_2_), 27.7 (CH_2_), 47.8 (CH_2_), 48.1 (CH_2_), 64.7 (CH_2_), 112.4 (CH), 118.9 (CH), 120.4 (CH), 120.5 (CH), 127.5 (CH), 134.0 (CH), 156.5 (C); IR (NaCl) υ_max _3132 (C-H Ar), 1599–1471 (C=C), 1165 (C-N), 1085 (C-O) cm^−1^; LCMS (M^+^)-SCN^−^ 287.3 found for C_18_H_27_N_2_O^+^.

*1-Pentyl-3-(4-phenoxybutyl)-1H-imidazol-3-ium nitrate* (**30**). ^1^H-NMR (CDCl_3_, 400 MHz): δ = 0.76 (t, *J* = 7.2 Hz, 3H), 1.20–1.37 (m, 4H), 1.70–1.79 (m, 4H), 2.03 (quint, *J* = 7.6 Hz, 2H), 3.88 (t, *J* = 7.6 Hz, 2H), 4.17 (t, *J* = 7.2 Hz, 2H), 4.34 (t, *J* = 7.6 Hz, 2H), 6.74–6.83 (m, 3H, Ar-H), 7.12–7.16 (m, 2H, Ar-H), 7.44–7.57 (m, 2H, Ar-H), 10.07 (s, 1H, Ar-H); ^13^C-NMR (CDCl_3_, 100 MHz): δ = 13.7 (CH_3_), 21.9 (CH_2_), 25.8 (CH_2_), 27.2 (CH_2_), 28.1 (CH_2_), 29.8 (CH_2_), 49.5 (CH_2_), 49.9 (CH_2_), 66.7 (CH_2_), 114.3 (CH), 120.7 (CH), 122.2 (CH), 122.5 (CH), 129.4 (CH), 136.6 (CH), 158.5 (C); IR (NaCl) υ_max _3130 (C-H Ar), 1599–1471 (C=C), 1163 (C-N), 1082 (C-O) cm^−1^; LCMS (M^+^)-NO_3_^−^ 287.3 found for C_18_H_27_N_2_O^+^.

*1-Hexyl-3-(4-phenoxybutyl)-1H-imidazol-3-ium tetrafluoroborate* (**31**). ^1^H-NMR (CDCl_3_, 400 MHz): δ = 0.98 (t, *J* = 7.2 Hz, 3H), 1.18 (quint, *J* = 7.6 Hz, 2H), 1.43–1.45 (m, 4H), 1.73 (quint, *J* = 7.6 Hz, 2H), 1.81 (quint, *J* = 7.6 Hz, 2H), 2.00 (quint, *J* = 7.6 Hz, 2H), 4.00 (t, *J* = 7.6 Hz, 2H), 4.31 (quint, *J* = 7.2 Hz, 2H), 4.50 (t, *J* = 7.6 Hz, 2H), 6.91–6.95 (m, 3H, Ar-H), 7.26–7.31 (m, 2H, Ar-H), 7.97–8.01 (m, 2H, Ar-H), 9.32 (s, 1H, Ar-H); ^13^C-NMR (CDCl_3_, 100 MHz): δ = 14.1 (CH_3_), 22.8 (CH_2_), 25.0 (CH_2_), 27.4 (CH_2_), 27.5 (CH_2_), 32.8 (CH_2_), 34.1 (CH_2_), 44.3 (CH_2_), 48.5 (CH_2_), 66.8 (CH_2_), 114.5 (CH), 120.4 (CH), 122.3 (CH), 122.5 (CH), 130.0 (CH), 136.2 (CH), 158.7 (C); ^19^F-NMR (CDCl_3, _376.5 MHz): δ = −148.25; ^11^B-NMR (CDCl_3, _128 MHz): δ = −1.26; IR (NaCl) υ_max _3129 (C-H Ar), 1602–1474 (C=C), 1165 (C-N), 1082 (C-O) cm^−1^; LCMS (M^+^)-BF_4_^−^ 301.3 found for C_19_H_29_N_2_O^+^.

*1-Hexyl-3-(4-phenoxybutyl)-1H-imidazol-3-ium hexafluorophospate* (**32**). ^1^H-NMR (CDCl_3_, 400 MHz): δ = 0.85 (t, *J* = 7.2 Hz, 3H), 1.12 (quint, *J* = 7.6 Hz, 2H), 1.25–1.34 (m, 4H), 1.79–1.88 (m, 4H), 2.10 (quint, *J* = 7.6 Hz, 2H), 3.97 (t, *J* = 7.6 Hz, 2H), 4.18 (t, *J* = 7.2 Hz, 2H), 4.33 (t, *J* = 7.6 Hz, 2H), 6.83–6.91 (m, 3H, Ar-H), 7.21–7.25 (m, 2H, Ar-H), 7.42–7.51 (m, 2H, Ar-H), 9.24 (s, 1H, Ar-H); ^13^C-NMR (CDCl_3_, 100 MHz): δ = 11.9 (CH_3_), 20.0 (CH_2_), 23.9 (CH_2_), 25.4 (CH_2_), 26.4 (CH_2_), 27.8 (CH_2_), 32.2 (CH_2_), 45.9 (CH_2_), 48.2 (CH_2_), 64.8 (CH_2_), 112.5 (CH), 119.0 (CH), 120.5 (CH), 120.6 (CH), 127.6 (CH), 134.2 (CH), 156.7 (C); ^19^F-NMR (CDCl_3, _376.5 MHz): δ = −71.10; ^31^P NMR (CDCl_3, _162 MHz): δ = −144.16 (sep *, J =* 712.8 Hz); IR (NaCl) υ_max _3133 (C-H Ar), 1599–1471 (C=C), 1165 (C-N), 1083 (C-O) cm^−1^; LCMS (M^+^)- PF_6_^−^ 301.3 found for C_19_H_29_N_2_O^+^.

*1-Hexyl-3-(4-phenoxybutyl)-1H-imidazol-3-ium trifluroacetate* (**33**). ^1^H-NMR (CDCl_3_, 400 MHz): δ = 0.79 (t, *J* = 7.2 Hz, 3H), 1.07 (quint, *J* = 7.6 Hz, 2H), 1.20–1.29 (m, 4H), 1.75–1.84 (m, 4H), 2.05 (quint, *J* = 7.6 Hz, 2H), 3.92 (t, *J* = 7.6 Hz, 2H), 4.13 (t, *J* = 7.2 Hz, 2H), 4.28 (t, *J* = 7.6 Hz, 2H), 6.79–6.87 (m, 3H, Ar-H), 7.17–7.23 (m, 2H, Ar-H), 7.38–7.47 (m, 2H, Ar-H), 10.15 (s, 1H, Ar-H); ^13^C-NMR (CDCl_3_, 100 MHz): δ = 13.0 (CH_3_), 21.1 (CH_2_), 24.0 (CH_2_), 26.5 (CH_2_), 27.6 (CH_2_), 28.9 (CH_2_), 33.4 (CH_2_), 47.0 (CH_2_), 49.3 (CH_2_), 65.9 (CH_2_), 113.6 (CH), 120.1 (CH), 121.6 (CH), 121.7 (CH), 128.7 (CH), 135.3 (CH), 157.8 (C); ^19^F-NMR (CDCl_3_, 376.5 MHz): δ = −75.41 ;IR (NaCl) υ_max _3133 (C-H Ar), 1599–1472 (C=C), 1162 (C-N), 1080 (C-O) cm^−1^; LCMS (M^+^)-CF_3_CO_2_^−^ 301.3 found for C_19_H_29_N_2_O^+^.

1-Hexyl-3-(4-phenoxybutyl)-1H-imidazol-3-ium dicyanoamine (**34**). ^1^H-NMR (CDCl_3_, 400 MHz): δ = 0.76 (t, *J* = 7.2 Hz, 3H), 1.04 (quint, *J* = 7.6 Hz, 2H), 1.20–1.37 (m, 4H), 1.70–1.79 (m, 4H), 2.03 (quint, *J* = 7.6 Hz, 2H), 3.88 (t, *J* = 7.6 Hz, 2H), 4.17 (t, *J* = 7.2 Hz, 2H), 4.34 (t, *J* = 7.6 Hz, 2H), 6.74–6.83 (m, 3H, Ar-H), 7.12–7.16 (m, 2H, Ar-H), 7.44–7.57 (m, 2H, Ar-H), 10.07 (s, 1H, Ar-H); ^13^C-NMR (CDCl_3_, 100 MHz): δ = 13.7 (CH_3_), 21.9 (CH_2_), 25.8 (CH_2_), 27.2 (CH_2_), 28.1 (CH_2_), 29.8 (CH_2_), 35.4 (CH_2_), 49.5 (CH_2_), 49.9 (CH_2_), 66.7 (CH_2_), 114.3 (CH), 120.7 (CH), 122.2 (CH), 122.5 (CH), 129.4 (CH), 136.6 (CH), 158.5 (C);IR (NaCl) υ_max _3133 (C-H Ar), 1599–1471 (C=C), 1165 (C-N), 1082 (C-O) cm^−1^; LCMS (M^+^)-(CN)_2_N^−^ 301.3 found for C_19_H_29_N_2_O^+^.

*1-Hexyl-3-(4-phenoxybutyl)-1H-imidazol-3-ium thiocyanate* (**35**). ^1^H-NMR (CDCl_3_, 400 MHz): δ = 0.82 (t, *J* = 7.2 Hz, 3H), 1.10 (quint, *J* = 7.6 Hz, 2H), 1.21–1.29 (m, 4H), 1.74–1.82 (m, 4H), 2.05 (quint, *J* = 7.6 Hz, 2H), 3.93 (t, *J* = 7.6 Hz, 2H), 4.18 (t, *J* = 7.2 Hz, 2H), 4.34 (t, *J* = 7.6 Hz, 2H), 6.80–6.89 (m, 3H, Ar-H), 7.18–7.22 (m, 2H, Ar-H), 7.38–7.48 (m, 2H, Ar-H), 10.02 (s, 1H, Ar-H); ^13^C-NMR (CDCl_3_, 100 MHz): δ = 11.9 (CH_3_), 20.1 (CH_2_), 23.9 (CH_2_), 25.4 (CH_2_), 26.3 (CH_2_), 28.0 (CH_2_), 34.0 (CH_2_), 47.7 (CH_2_), 48.1 (CH_2_), 64.9 (CH_2_), 112.5 (CH), 119.0 (CH), 120.2 (CH), 120.5 (CH), 127.6 (CH), 135.1 (CH), 156.7 (C); IR (NaCl) υ_max _3128 (C-H Ar), 1602–1471 (C=C), 1164 (C-N), 1081 (C-O) cm^−1^; LCMS (M^+^)-SCN^ −^ 301.3 found for C_19_H_29_N_2_O^+^.

*1-Hexyl-3-(4-phenoxybutyl)-1H-imidazol-3-ium nitrate* (**36**). ^1^H-NMR (CDCl_3_, 400 MHz): δ = 0.85 (t, *J* = 7.2 Hz, 3H), 1.14 (quint, *J* = 7.6 Hz, 2H), 1.24–1.33 (m, 4H), 1.78–1.87 (m, 4H), 2.09 (quint, *J* = 7.6 Hz, 2H), 3.97 (t, *J* = 7.6 Hz, 2H), 4.18 (t, *J* = 7.2 Hz, 2H), 4.33 (t, *J* = 7.6 Hz, 2H), 6.82–6.90 (m, 3H, Ar-H), 7.20–7.24 (m, 2H, Ar-H), 7.41–7.50 (m, 2H, Ar-H), 9.59 (s, 1H, Ar-H); ^13^C-NMR (CDCl_3_, 100 MHz): δ = 11.8 (CH_3_), 19.9 (CH_2_), 23.8 (CH_2_), 25.3 (CH_2_), 26.2 (CH_2_), 27.7 (CH_2_), 34.2 (CH_2_), 47.8 (CH_2_), 48.1 (CH_2_), 64.7 (CH_2_), 112.4 (CH), 118.9 (CH), 120.4 (CH), 120.5 (CH), 127.5 (CH), 134.0 (CH), 156.5 (C);IR (NaCl) υ_max _3131 (C-H Ar), 1598–1470 (C=C), 1164 (C-N), 1081 (C-O) cm^−1^; LCMS (M^+^)-NO_3_^−^ 301.3 found for C_19_H_29_N_2_O^+^.

### 3.4. Determination of Minimum Inhibitory Concentrations

Minimum inhibitory concentrations (MICs) were determined using the broth microdilution method based on recommended protocolemployed by the Clinical and Laboratory Standards Institute [41]. Tested compounds were dissolved in sterile, distilled water and diluted to a final concentration of 512 µg/mL in Mueller-Hinton broth (Becton Dickinson, Franklin Lakes, NJ, USA) [42]. Two-fold serially diluted test compounds were dispensed into each of the 96 wells of a standard microdilution plates. The direct colony suspension method was used for inoculum preparation. Bacterial suspension was prepared by direct transfer of colonies from 24 h agar plates to Mueller Hinton broth. Bacterial suspensions were adjusted using bacterial counting chamber to contain approximately 1 × 10^8^ CFU/mL. A 50 µL volume of each bacterial suspension was mixed with 50 µL serially diluted tested compound in 96 microdilution plate according to the microdilution method [43]. Uninoculated wells were prepared as control samples.Plates were incubated at 35 °C for 24 h. The minimum (inhibitory) bactericidal concentration was defined as the lowestconcentration of test compound producing no visible growth. Confirmation for MIC, was achieved by transfer of aliquots from wells containing no growth on to nutrient agar plates and tested for colony formation upon subculturing. Given values of obtained MIC values are means of three independent experiments.

## 4. Conclusions

In summary, new eco-friendly 1-alkyl-3-(4-phenoxybutyl) imidazolium-based ionic liquids (ILs) derivatives were prepared by using ultrasound irradiation. Many advantages for the ultrasound assisted synthesis compared with the standard methods have been recorded. The ILs studied displayed a very promising antimicrobial activity. Their activities are greatly affected by the alkyl chain length. The study of the physicochemical properties, water content, biodegradability and solubility of the newly synthesized ionic liquids will be will be discussed in details in the forthcoming paper.
